# Predictors of surgical outcome in thoracic ossification of the ligamentum flavum: focusing on the quantitative signal intensity

**DOI:** 10.1038/srep23019

**Published:** 2016-03-10

**Authors:** JingTao Zhang, LinFeng Wang, Jie Li, Peng Yang, Yong Shen

**Affiliations:** 1 Department of Spinal Surgery, The Third Hospital of Hebei Medical University, Shijiazhuang, Hebei, People’s Republic of China

## Abstract

The association between intramedullary increased signal intensity (ISI) on T2-weighted magnetic resonance imaging (MRI) and surgical outcome in thoracic ossification of the ligamentum flavum (OLF) remains controversial. We aimed to determine the impact of signal change ratio (SCR) on thoracic OLF surgical outcomes. We retrospectively reviewed 96 cases of thoracic OLF surgery and investigated myelopathy severity, symptom duration, MRI and computed tomographic findings, surgical technique and postoperative recoveries. Surgical outcomes were evaluated according to the modified Japanese Orthopaedic Association (JOA) score and recovery rate. JOA recovery rate <50% was defined as a poor surgical outcome. By multivariate logistic regression analysis, we identified risk factors associated with surgical outcomes. Forty patients (41.7%) had a recovery rate of <50%. In receiver operating characteristic (ROC) curves, the optimal preoperative SCR cutoff value as a predictor of poor surgical outcome was 1.54. Multivariate logistic regression analysis revealed that a preoperative SCR ≥1.54 and symptom duration >12 months were significant risk factors for a poor surgical outcome. These findings suggest that preoperative SCR and duration of symptoms were significant risk factors of surgical outcome for patients with thoracic OLF. Patients with preoperative SCR ≥1.54 can experience poor postoperative recovery.

Thoracic ossification of the ligamentum flavum (OLF) is a relatively rare cause of myelopathy that generally requires surgical treatment owing to its progressive nature and its poor response to conservative therapy. Reportedly, the prevalence of OLF ranges from 3.8% to 26%^1^,^2^. Patients with thoracic OLF have various symptoms, such as sensory abnormality of the trunk or lower extremities, gait disturbance, and urinary dysfunction^3^. Although decompressive surgery is an available treatment option for this disease, the surgical outcome is not always satisfactory. Prognostic guidelines are still unclear and it is very difficult for the surgeon to predict postoperative recovery.

Magnetic resonance imaging (MRI) is a valuable tool before surgical decompression because it allows the visualisation not only of the magnitude of spinal cord compression but also of intramedullary signal intensity. The presence of intramedullary increased signal intensity (ISI) on T2-weighted imaging (WI) in patients with thoracic OLF reflects chronic spinal cord compression. However, the significance of ISI on T2-WI for postsurgical prognosis remains controversial^4^,^5^,^6^,^7^,^8^,^9^,^10^,^11^,^12^,^13^,^14^,^15^. Definitive information is not available because most descriptions of signal changes are qualitative in nature. Wang *et al.*^16^ first used signal change ratio (SCR) as a quantifiable measure of signal intensity in cervical compressive myelopathy. This quantitative method may also be used to evaluate the signal change of the spinal cord in thoracic OLF. The purpose of this study was to assess the risk factors associated with poor surgical outcome, particularly the predictive value of quantitative SCR, after surgical treatment for thoracic OLF and ascertain the crucial determinants of surgical outcome using statistical analyses.

## Methods

### Ethics statement

The study was approved by Ethics Committee of the Third Hospital of Hebei Medical University in China and patient consent was not required due to the retrospective nature of this study. Patient information was anonymized and re-identified prior to analysis. The methods were carried out in accordance with the approved guidelines.

### Study design and patient population

This retrospective study included data of patients who underwent surgery for thoracic myelopathy secondary to OLF at the Department of Spinal Surgery, the Third Hospital of Hebei Medical University in China, between January 1997 and December 2012. Exclusion criteria were previous thoracic surgery, ventral compressive lesion, such as thoracic ossification of the posterior longitudinal ligament or thoracic disc herniation, vitamin B deficiency, other neuromuscular disorders and concomitant cervical or lumbar lesions before their OLF surgery or during follow-up. A total of 96 patients with thoracic OLF were ultimately included in this study. There were 66 men and 30 women, ranging in age from 42 to 80 years, with a mean age of 65 years. The mean follow-up period was 5.2 years, ranging from 2 to 11 years. Diagnoses were confirmed by neurological examinations and imaging studies, including routine radiographs, computed tomography (CT) and MRI.

Surgical indications were progressive thoracic myelopathy and dorsal pain. We performed total laminectomy and resection of the OLF at the involved levels. The dura beneath the OLF was removed and duraplasty was performed if the dura was adhered to OLF and could not be separated. Posterior fusion with instrumentation was carried out when laminectomy was performed in three or more levels.

### Neurological assessment

The preoperative and postoperative neurological function at 2 years of follow-up was assessed using a modified Japanese Orthopaedic Association (JOA) scoring system (Table 1). This scoring system was derived from the JOA scores for cervical myelopathy by eliminating the motor and sensory scores for the upper extremities^17^. The maximum score is 11, and it indicates normal function. Postoperative improvement of symptoms was estimated on the basis of the recovery rate (RR) = (postoperative JOA score−preoperative JOA score)/(11−preoperative JOA score) × 100%. A score of 75 to 100% was designated as excellent, 50 to 74% as good, 25 to 49% as fair and 0 to 24% as poor. Therefore, in this study, we defined a poor surgical outcome as a recovery rate less than 50%.

### Radiographic assessment

We used the CT axial classification developed by Sato *et al.*^18^ to evaluate CT images. We found the following four types of OLF in our subjects: lateral, extended and enlarged, fused, and tuberous. The lateral type lesion showed OLF only at the facet joint capsule; the extended and enlarged type showed OLF extending to the lamina with antero-medial enlargement; the fused type showed thickened bilateral OLF fused at the midline; and the tuberous type showed fused OLF growing anteriorly.

All patients underwent preoperative high-resolution MRI with a 1.5-T system (Magnetom Symphony, Siemens Medical Solutions, Malvern, PA, USA). The MRIs of the spinal cord were obtained using a spin echo sequence system for T1-WI and a fast spin echo sequence system for T2-WI. The high-signal intensity values of the spinal cord on sagittal T2-WIs were obtained, and the regions of interest (ROIs) were taken by 0.05 cm^2^. The normal spinal cord signal intensity values on sagittal T2-WIs were obtained at C7-T1 disc level, and the ROIs were taken by 0.3 cm^2^. If no intramedullary ISI was noted on T2-WIs, the ROIs were taken by 0.05 cm^2^ of the severely compressed cord. The SCR was defined as the signal intensity at the level of ISI or severely compressed cord (in cases with no ISI) divided by the signal intensity at C7-T1 disc level. The signal intensity value was measured on the MRI workstation, and the SCR was calculated. The selection of the ROIs was based on the balance of a number of factors. For instance, an extremely large area would not hold all patients in the group, whereas an extremely small area would jeopardise the accuracy of the signal intensity value.

### Statistical analysis

Descriptive analysis of the patient population was conducted using means and standard deviations (SD) for continuous variables and frequencies and percentages for categorical variables. Pearson’s correlation coefficient was used to check the correlation between preoperative SCR and JOA recovery rate. Receiver operating characteristic (ROC) curve was constructed to evaluate the sensitivity and specificity of preoperative SCR in predicting poor surgical outcome postoperatively. Inferential statistics were performed to assess the association between the independent risk factors and recovery status using independent Student’s t-tests for continuous variables and Chi-square or Fisher’s exact tests to analyse categorical variables. Multivariate logistic regression analysis was also performed in order to control for potential confounding variables with the end point of ‘poor surgical outcome’. Adjusted odds ratios with 95% confidence intervals (CI) were presented with their respective p-values. Factors with a p-value of less than 0.20 in univariate analysis were entered into the multivariate logistic model. A value of P < 0.05 was considered to represent a statistically significant difference. All analyses were performed using SPSS software (version 21.0; SPSS Inc., Chicago, IL, USA).

## Results

### Preoperative and postoperative JOA scores

For all patients, the mean JOA score was 5.5 points preoperatively, and 8.0 points at 2 years postoperatively, yielding a mean recovery rate of 45.8%. Thus, a statistically significant improvement in the JOA score was obtained at the 2-year follow-up (p < 0.05). Fifty-six patients had good surgical outcomes, with recovery rates greater than or equal to 50%, while 40 patients had poor surgical outcomes with recovery rates less than 50%. The average preoperative JOA scores for the good recovery group and poor recovery group were not significantly different (5.7 vs 5.4, p = 0.436). The postoperative JOA score was significantly improved in the good recovery group compared with the poor recovery group at 2 years after surgery (9.2 vs 7.2, p < 0.001).

### SCR and neurological improvement

**Figure 1:**
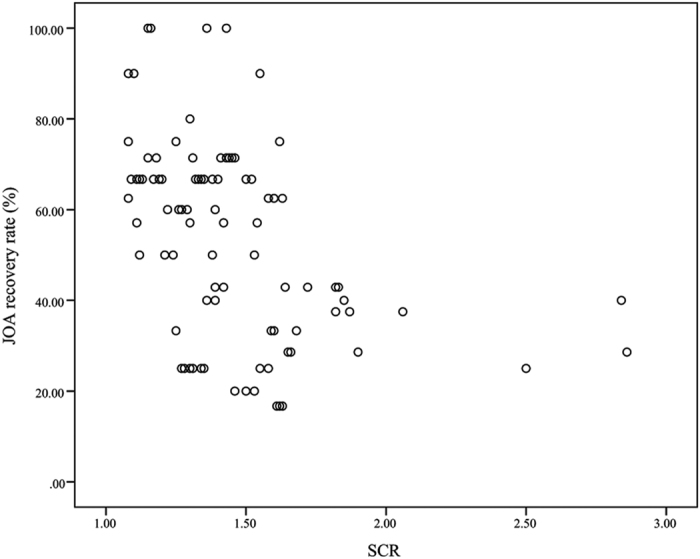
Correlation between preoperative SCR and JOA recovery rate after operation (r = −0.422; p < 0.001).

**Figure 2:**
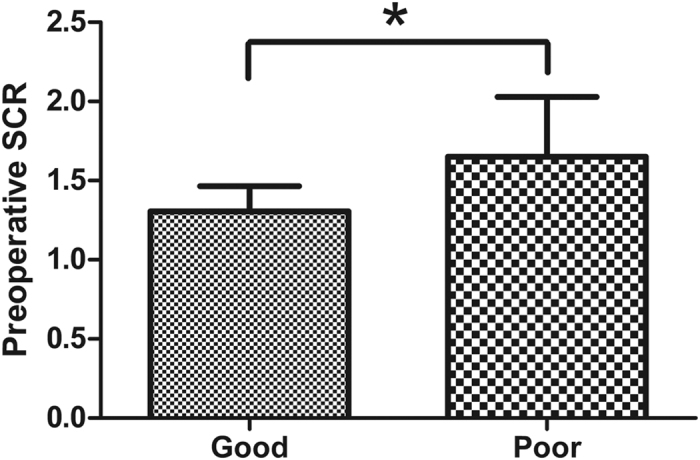
The preoperative SCR in the good recovery group (n = 56) and poor recovery group (n = 40). Values presented are mean ± SD. ^*^p < 0.001. Student’s t test.

**Figure 3:**
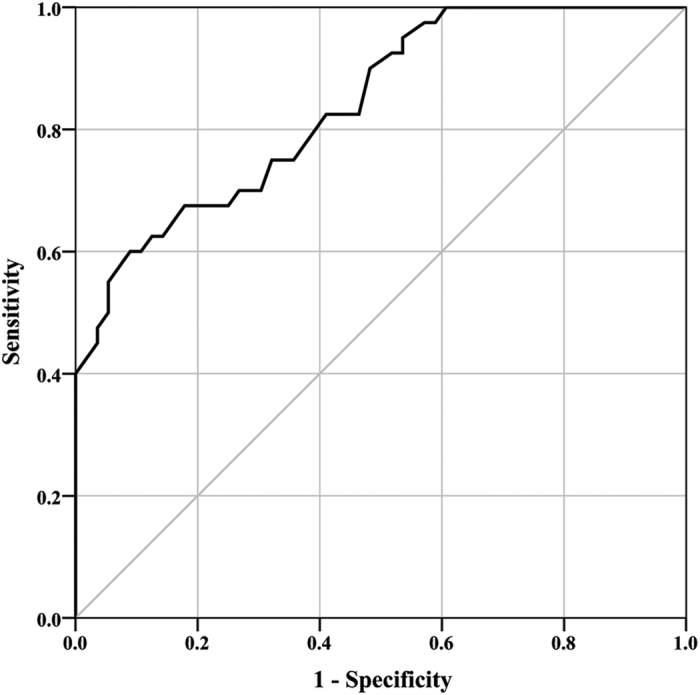
In receiver operating characteristic curves, the optimal cutoff value of preoperative SCR is shown for prediction of a poor surgical outcome.

For all patients, preoperative SCR on T2-WI ranged from 1.08 to 2.86. Figure 1 demonstrated that preoperative SCR correlated with (r = −0.422; p < 0.001) JOA recovery rate after operation. The preoperative SCR was significantly greater in the poor recovery group than in the good recovery group (1.65 vs 1.31, p < 0.001; Fig. 2). To further clarify the importance of preoperative SCR on T2-WI, we calculated a cutoff value for SCR. The ROC curve analysis showed that 1.54 was the cutoff value to maximise the power of preoperative SCR as a predictor of poor surgical outcome (Fig. 3). The area under the ROC curve of preoperative SCR for predicting poor surgical outcome was 0.839 (95% CI, 0.761–0.917; p < 0.001).

### Comparison of clinical and radiological data between groups

Demographic characteristics, clinical and radiological data are listed in Table 2. Univariate analysis revealed that age category (p = 0.022), symptom duration category (p = 0.006) and preoperative SCR category (p = 0.004) were significantly associated with poor surgical outcome, but sex (p = 0.823) and diabetes mellitus (p = 0.443) were not. There were no significant differences between groups in radiological data, such as OLF level (p = 0.452), number of OLF levels (p = 0.613), ossification of dura mater (p = 0.679) and CT axial classification (p = 0.716). Regarding operative factors, there was no significant difference in the type of surgical technique between the two groups (p = 1.000). The operation-related complications were also compared. Hematoma occurred in 3.6% of subjects in good recovery group and in 5.0% of subjects in poor recovery group (p = 1.000). Cerebrospinal fluid (CSF) leakage occurred in 5.4% of subjects in good recovery group and in 10.0% of subjects in poor recovery group (p = 0.446). Wound infection occurred in 0% of subjects in good recovery group and in 2.5% of subjects in poor recovery group (p = 0.417).

### Factors associated with poor surgical outcome

In multivariate logistic regression analysis, duration of symptoms (odds ratio; 1 for symptom duration less than 6 months versus 3.968 [95% CI; 1.422–11.067, p = 0.008] for symptom duration greater than 12 months), and preoperative SCR (odds ratio; 1 for less than 1.54 versus 2.860 [95% CI; 1.174–6.967, p = 0.021] for 1.54 or greater) were identified as independent predictors of poor improvement according to the JOA scores (Table 3).

## Discussion

The elucidation of factors that contribute to prognosis of patients with thoracic OLF has been investigated by several groups^4^,^5^,^6^,^7^,^8^,^9^,^10^,^11^,^12^,^13^,^14^,^15^,^17^,^19^,^20^,^21^,^22^. Recognition of the best timing for surgery to ensure neurological improvement is an important clinical issue. Thus far, numerous factors have been reported to affect postoperative outcomes in patients with thoracic OLF. Age^23^, duration of myelopathic symptoms^6^,^10^,^19^,^21^,^23^,^24^,^25^,^26^, levels of OLF^27^, CT axial classification^6^,^9^,^21^, signal intensity changes on preoperative MRI^4^,^5^,^6^,^7^,^8^, and preoperative JOA score^6^,^11^,^12^,^13^,^21^ have been considered key predictors. However, the list of predictive factors differs according to researchers, and the prognostic significance of these factors remains controversial.

The ISI of the spinal cord on T2-WI is often observed in patients with thoracic OLF, and various authors have speculated on its histopathologic significance and impact on surgical outcome. Studies have reported that ISI on T2-WI indicates local pathologic changes in the spinal cord, and patients with thoracic OLF and high-signal intensity on T2-WI usually have a poor prognosis even after surgical intervention^4^,^5^,^6^,^7^,^8^. Al-Mefty *et al.*^28^ reported that spinal cord ISI on T2-WI reflected myelomalacia, and that low-signal changes on T1-WI indicated cystic necrosis or secondary syrinx. Sanghvi *et al.*^7^ indicated that preoperative intramedullary signal size in T2-WI was a major factor affecting recovery in a large group of patients with thoracic OLF. Despite such evidence, many studies found no correlation between surgical outcome and intramedullary ISI on T2-WI^9^,^10^,^11^,^12^,^13^,^14^,^15^.

What is the reason for this discrepancy? The following may explain this phenomenon. Although MRI provides high specificity in the assessment of both morphologic and intramedullary changes of the thoracic spinal cord, it is almost impossible to estimate potential recovery of the thoracic spinal cord on preoperative MRI without appropriate quantitative analysis. We consider that ISI is a wide-ranging variant that usually encompasses many levels of actual severity. If we only were to evaluate patients according to ISI, the results would be biased because of possible judgment errors by each investigator. Considering these factors, in this study, signal change was evaluated according to a quantitative approach. SCR was the quantitative method used to assess the changes of signal intensity, as described in a previous study^16^. In the present study, we calculated that the optimal cutoff value of preoperative SCR as a predictor of poor postoperative outcome was 1.54. Specifically, the odds of a poor outcome were 2.86 times greater for patients with SCR greater or equal to 1.54 than for those with SCR less than 1.54. As far as we know, this study is the first to report that a preoperative SCR greater than or equal to 1.54 can predict poor recovery after surgical treatment for patients with thoracic OLF. Therefore, we consider that recognition of SCR contributes significantly to the determination of the most appropriate timing for surgery.

Moreover, we found that duration of symptoms was another independent predictive factor affecting postoperative surgical outcome. The odds of a poor outcome were 3.97 times greater for patients with more than 12 months of symptom duration than for those with less than 6 months of symptom duration. The rationale is that long-standing and chronic compression of the spinal cord may lead to irreversible damage due to demyelination and necrosis of the grey matter. Therefore, to achieve the best results, surgical intervention should be undertaken as early as possible. The results of the univariate analysis showed that patients in the poor outcome group were significantly older than those in the good outcome group (p = 0.022). Age was also selected to multivariate logistic analysis. However, after adjustments for SCR and duration of symptoms, age did not significantly correlate with poor outcome. Although most surgeons will not discriminate on the basis of age, they should be aware that elderly patients may experience poor neurological recovery. In our study, patient demographic variables, such as sex, diabetes mellitus and preoperative JOA score did not influence the outcome of the surgical intervention. The CT axial classification, levels of OLF and the number of OLF segments did not correlate with surgical outcome. It is also uncertain whether these factors are predictive of surgical outcome, as our findings are inconsistent with the results of previous studies. It remains to be seen, however, whether these factors are truly unrelated to surgical outcome. It is possible that statistical significance was not reached in this study because of the limited number of thoracic OLF patients and/or the different statistical tests used across the different studies.

There are several limitations that need to be considered in our study. First, this was a single-centre study and involved only a small number of thoracic OLF patients. Second, the period of the greatest extent of recovery after surgery for thoracic OLF is unclear, evaluation at 2 years postoperatively may not be sufficient. Third, each high-resolution MRI system has different characteristic and working parameters, which may explain the differences between our results and those of previous studies. Therefore, these limitations suggest that our findings require further validation of these results in larger patient samples.

## Conclusions

The quantification of signal changes in patients with thoracic OLF was used in the present study to assess the impact of intramedullary signal change on the surgical outcome. Preoperative SCR on T2-WI could be potentially useful for the prediction of the surgical outcome in patients with thoracic OLF. Patients with preoperative SCR greater than or equal to 1.54 are likely to experience poor postoperative recovery.

## Additional Information

**How to cite this article**: Zhang, J.T. *et al.* Predictors of surgical outcome in thoracic ossification of the ligamentum flavum: focusing on the quantitative signal intensity. *Sci. Rep.* **6**, 23019; doi: 10.1038/srep23019 (2016).

## Figures and Tables

**Table 1:** Modified Japanese Orthopaedic Association (JOA) scoring system.

Categories	Score (Points)
Motor function: lower extremity	
Impossible to walk	0
Need a cane or aid on flat ground	1
Need aid only on stairs	2
Possible to walk without any aid, but slow manner	3
Normal	4
Sensory function: lower extremity	
Apparent sensory disturbance	0
Minimal sensory disturbance	1
Normal	2
Sensory function: Trunk	
Apparent sensory disturbance	0
Minimal sensory disturbance	1
Normal	2
Bladder function	
Urinary retention or incontinence	0
Severe dysuria (sense of retention, staining)	1
Slight dysuria (pollakisuria, retardation)	2
Normal	3
Total score	11

**Table 2:** Comparison of patient characteristics between good and poor surgical outcome groups.

Variable	Good (n = 56)	Poor (n = 40)	P-value
Age at operation (yr)			
<60	16	7	0.022
60–70	26	12	
>70	14	21	
Male sex (n, %)	39 (69.6%)	27 (67.5%)	0.823
Diabetes mellitus (n, %)	15 (26.8%)	8 (20.0%)	0.443
Duration of symptoms (mo)			
<6	28	9	0.006
6–12	13	8	
>12	15	23	
Preoperative JOA score	5.7 ± 1.7	5.4 ± 1.4	0.436
Postoperative JOA score	9.2 ± 0.8	7.2 ± 0.9	0.000
Preoperative SCR			
<1.54	39	16	0.004
≥1.54	17	24	
OLF level			
T1-T5	4	3	0.452
T5-T9	3	5	
T9-T12	49	32	
Number of OLF Levels			
1	33	20	0.613
2	17	16	
>2	6	4	
Ossification of dura mater (n, %)	27(48.2%)	21(52.5%)	0.679
CT axial classification			
Lateral	10	4	0.716
Extended and enlarged	12	10	
Fused	30	22	
Tuberous	4	4	
Surgical technique			
Laminectomy	50	36	1.000
Posterior decompression and fusion	6	4	
Complications			
Hematoma	2/56(3.6%)	2/40(5.0%)	1.000
CSF leakage	3/56(5.4%)	4/40(10.0%)	0.446
Wound infection	0/56(0%)	1/40(2.5%)	0.417

JOA: Japanese Orthopaedic Association; SCR: signal change ratio; OLF: ossification of the ligamentum flavum; CT: computed tomography; CSF: cerebrospinal fluid; mo: month; yr: year

**Table 3:** Risk factors for poor postoperative outcome: multiple logistic regression analysis.

Variable	Odds Ratio (95% Confidence Interval)	P-value
Age at operation (yr)		
<60	1	
60–70	1.352 (0.415–4.403)	0.616
>70	2.244 (0.682–7.384)	0.183
Duration of symptoms (mo)		
<6	1	
6–12	1.714 (0.519–5.655)	0.377
>12	3.968 (1.422–11.067)	0.008
Preoperative SCR		
<1.54	1	
≥1.54	2.860 (1.174–6.967)	0.021

SCR: signal change ratio; mo: month; yr: year.
